# Analysis of Giant Asymptomatic Thymic Fibrolipoadenoma Using Dual-Layer Detector Spectral CT

**DOI:** 10.1148/rycan.250050

**Published:** 2025-06-20

**Authors:** Haojie Zhang, Zhigang Zhou, Songzi Kou, Yuhan Zhou

**Affiliations:** ^1^Department of Radiology, The First Affiliated Hospital of Zhengzhou University, No. 1 Jianshe E Rd, Zhengzhou 450000, China; ^2^Department of Pathology, The First Affiliated Hospital of Zhengzhou University, Zhengzhou, China

**Keywords:** CT, CT-Dual Energy, Thymus Gland, Tumor

*Supplemental material is available for this article.*

A 28-year-old female patient was admitted to the hospital with suspected mediastinal mass due to absent breath sounds over the left lower lung field and dullness to percussion at routine physical examination without any chest symptoms, prompting referral for chest CT. The spectral CT-derived venous fused image (40-keV virtual monoenergetic and iodine density) showed a large heterogeneous mass extending into both sides of the thoracic cavity, clearly revealing the composition of the mass and the supplying blood vessel (Fig 1A, Movie). The spectral attenuation curve, histogram, and scatterplot confirmed the homogeneity among the low-attenuation fatlike components, patchy solid components, and high iodine-containing cordlike dense structures, respectively (Fig 1B). Postoperative pathologic results confirmed the diagnosis of thymic lipofibroadenoma, with the fatty and fibrous components within the lesion matching the spectral CT findings (Fig 2).

**Figure 1:**
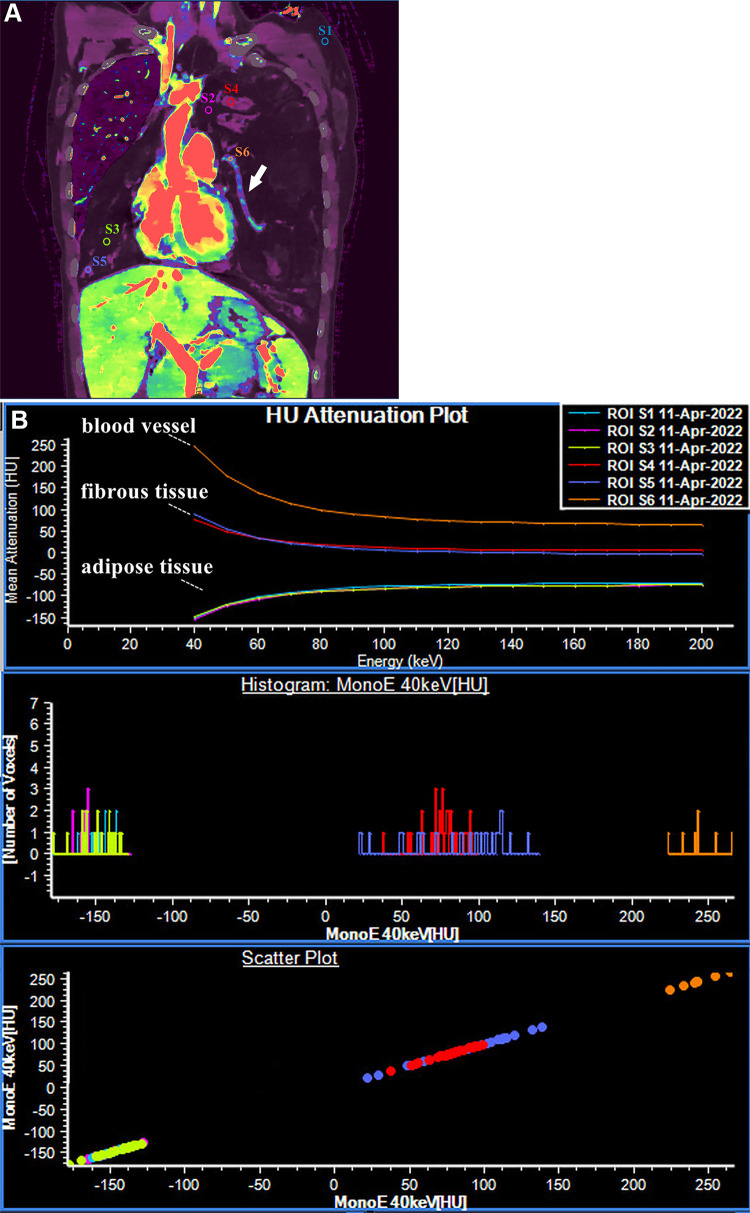
Spectral CT images in a 28-year-old female patient with thymic fibrolipoadenoma. **(A)** Venous phase fused image (40-keV virtual monoenergetic imaging and iodine density) clearly delineates the relationship between the mass, lungs, and heart, as well as the internal composition of the mass and its blood vessel (white arrow). **(B)** The spectral curves show that the low-attenuation components (ROI: S2, S3) on both sides are consistent with that of subcutaneous fat (ROI: S1), and the spectral curve slopes of the patchy solid components (ROI: S4, S5) on both sides are identical, suggesting a common origin. Additionally, the spectral curve slope of the cordlike dense structure (white arrow, **A**) is different from the other components, indicating that it is not part of the parenchymal fractions of the mass. HU = Hounsfield unit, ROI = region of interest.

**Movie:** Images from 40-keV monoenergetic contrast-enhanced CT during the venous phase, clearly demonstrating a large heterogeneous mass in the anterior mediastinum. The mass extends into both thoracic cavities, with more prominent involvement of the left side. The mass predominantly shows low attenuation, interspersed with linear, patchy, and nodular areas of solid tissue with mild enhancement. Compression of the lungs is evident, with bilateral pulmonary atelectasis due to the mass's large size. The arterial supply to the mass is from the brachiocephalic trunk (yellow arrow), and venous drainage occurs through the draining vein (red arrow) into the left brachiocephalic vein.

**Figure 2:**
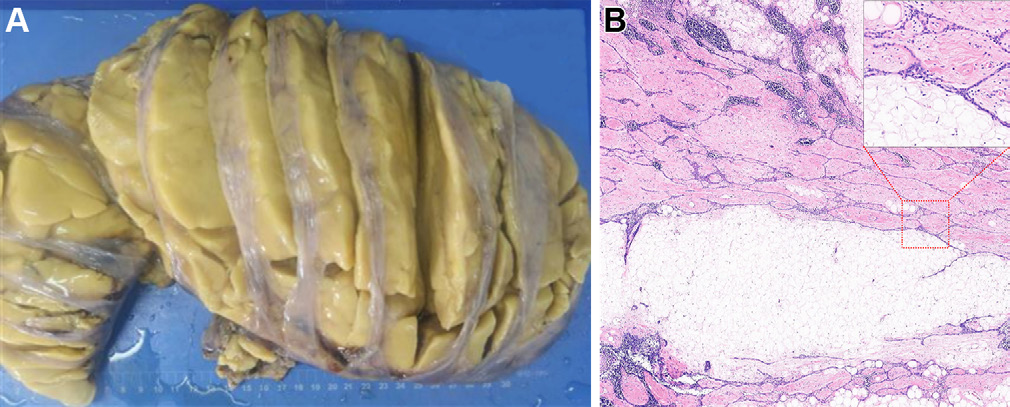
Gross pathologic findings in the same patient. **(A)** Photograph of gross pathologic specimen. Mass is firm with a yellow appearance and a fibrous thin membrane. **(B)** Photomicrograph of the surgical specimen shows mature fat, fibrous tissue, and figurate strands of thymic epithelial cells in the mass (hematoxylin-eosin stain; scale bar, 1.4 mm). Pathologic diagnosis of the giant mass was lipofibroadenoma of the thymus, which was consistent with spectral CT findings.

Thymic lipofibroadenoma is an extremely rare benign tumor, first reported in 2001 (1). The etiology and pathogenesis of this disease remain unclear, but it may be associated with type B1 thymoma (1,2). In this case, the spectral curves of each region consistently confirm the presence of mature adipose tissue, homologous fibrous tissue, and blood vessel. This noninvasive analysis, combined with pathologic features, facilitates a deeper understanding of mass characteristics, leading to more accurate diagnosis.

## Notes

**Keywords:** CT, CT-Dual Energy, Thymus Gland, Tumor
